# Transforming viticulture through genomic innovation and integrated pest management for sustainable grape production

**DOI:** 10.1038/s44383-026-00038-4

**Published:** 2026-08-04

**Authors:** Giorgio Gambino, Lara Agnoli, Walter Chitarra, Marc Dressler, Andreia Figueiredo, Mario de la Fuente, Gabriele Di Gaspero, Ludger Hausmann, Christos Karatzas, Benoit Laurent, Pere Mestre, Irene Perrone, Komlan Avia

**Affiliations:** 1 Institute for Sustainable Plant Protection, National Research Council (CNR-IPSP), Torino, Italy; 2 Burgundy School of Business, School of Wine & Spirits Business, CEREN EA 7477, Dijon, France; 3 Council for Agricultural Research and Economics Centre of Viticulture and Enology Research (CREA-VE), Conegliano, Italy; 4 University of Ludwigshafen, Winecampus, Ludwigshafen, Germany; 5 Grapevine Pathogen Systems Lab, Biosystems and Integrative Sciences Institute (BioISI), Science Faculty of Lisbon University, Lisbon, Portugal; 6 Spanish Wine Technology Platform - CEIGRAM-Universidad Politécnica de Madrid, Madrid, Spain; 7 Istituto di Genomica Applicata, Udine, Italy; 8 Julius Kühn Institute (JKI), Institute for Grapevine Breeding Geilweilerhof, Siebeldingen, Germany; 9 AGRENAOS Agricultural Advisory Services, Athens, Greece; 10 Institut Français de la Vigne et du Vin, Domaine de l’Espiguette, Le Grau-du-Roi, France; 11 INRAE, Université de Strasbourg, UMR SVQV, Colmar, France

**Keywords:** Biotechnology, Plant sciences

## Abstract

Climate change and intensifying disease pressure push viticulture beyond incremental adaptation, especially where grape production depends on repeated pesticide applications. Reducing chemical inputs requires an integrated strategy combining broader genetic diversity, conventional and genomic-assisted breeding, new genomic techniques, somaclonal and clonal variation, and cultivar-tailored IPM. The Horizon Europe projects Shield4Grape and GrapeBreed4IPM operationalize this vision across genetics, agronomy, socioeconomics, and policy, with success depending on governance, stewardship, and proportionate regulation.

## Introduction

Climate change and environmental degradation pose unprecedented challenges to global sustainability. Despite significant progress in agricultural technologies, consideration of ecological boundaries has not always been fully integrated into their development, resulting in resource depletion and biodiversity loss. The resulting environmental shifts threaten water and soil fertility, compromise agricultural productivity and food quality, and exacerbate the spread of plant pests and diseases^1^.

Grapevine (*Vitis* spp.) is among the world’s most valuable perennial fruit crops and is central to cultural heritage, landscape identity and rural economies. Viticulture has long shaped cultural landscapes and regional identity, particularly in Europe^2^. The *Vitis* genus is characterized by a high level of genetic diversity. Most cultivated grapevines belong to the Euvitis subgenus, which is organized into three major groups: the American and East Asian groups, which are the main sources of resistance to diseases such as powdery mildew (PM) and downy mildew (DM), caused respectively by *Erysiphe necator* and *Plasmopara viticola*; and the Eurasian group, made of a single species, *Vitis vinifera* L., which accounts for most of the world’s *Vitis* varieties^3^. Despite the rich genetic diversity within the *Vitis* genus, modern viticulture overwhelmingly depends on a narrow selection of *V. vinifera* cultivars^4^, with fewer than 10 varieties occupying more than 60% of vineyards in some top-producing countries and about one-third of vineyards worldwide^5,6^. This narrow exploitation of the available biodiversity constitutes a structural vulnerability of the sector: an effective response to today’s rapidly evolving biotic and abiotic pressures can only be built by mobilizing the full range of genetic resources offered by the *Vitis* genus^7^.

**Fig. 1:**
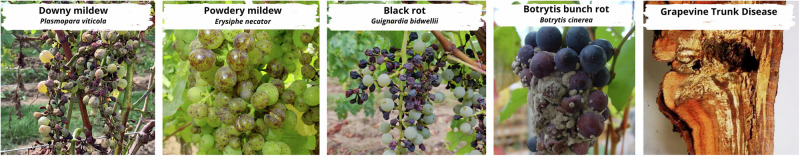
Main grapevine diseases targeted by Shield4Grape and GrapeBreed4IPM projects.

Viticulture and enology are vital components of the agro-industrial economy of several regions worldwide, yet they also account for some of the highest pesticide inputs in agriculture. This disparity is well illustrated by France, for example, where vineyards occupy just 3% of cultivated land yet receive about 20% of agricultural pesticides^8,9^. This heavy reliance stems primarily from the need to control pathogens and pests introduced from North America in the 19th century, which have caused persistent and severe disease outbreaks^10^. In particular, the most damaging and widely distributed fungal and oomycete diseases are PM and DM, whose management carries substantial economic and environmental costs^11–13^. Furthermore, additional fungal diseases have emerged as significant threats to grape yield and quality in recent years, including black rot^14,15^, Botrytis bunch rot^16,17^, and grapevine trunk diseases^18,19^ (Fig. 1), a challenge compounded by the limited availability of effective protection strategies. Although measurable progress has been made in reducing pesticide inputs^20^, viticulture’s continued heavy reliance on synthetic chemicals and copper-based products highlights the urgent need for more transformative approaches^21^.

The urgency to redesign viticulture in the face of global change is compounded by the complexity of the viticultural system itself. Vines are perennial, long-lived, and highly sensitive to environmental cues; their productivity and fruit quality are the outcome of decades of interaction among genotype, climate, soil, and management practices. These interactions underpin the concept of “terroir”, which integrates climatic, soil, topographic, and human factors to shape grapevine performance, wine typicity, and regional identity. Rather than being a static attribute, terroir reflects a dynamic equilibrium between genotype and environment, mediated by viticultural practices, and is therefore inherently sensitive to changing agro-climatic conditions^22,23^. Changes in temperature, rainfall patterns, and pathogen pressure can reverberate for years across vineyards, with cascading effects on yields, microbial ecology, and wine character. Climate change is already reshaping viticulture through rising temperatures, altered precipitation regimes, and a higher frequency of extreme events, including heatwaves and spring frosts. These changes are causing modifications in the phenology of the vines, with early flowering and ripening, and are modifying grape composition and wine typicity^24,25^. Within this complex system, rootstocks play a critical and often underappreciated role by mediating plant responses to soil and climatic conditions, and by modulating interactions between scion genotype and environment. Their contribution is increasingly recognized as a key component of viticulture adaptation to global change, particularly in shaping tolerance to abiotic stresses such as drought and heat, as well as influencing vine vigor and productivity^26^. Furthermore, climate-driven alterations in environmental conditions are influencing pathogen dynamics, potentially increasing disease pressure and changing the timing and intensity of epidemics. At the same time, there is a growing societal demand for agricultural systems that safeguard biodiversity and reduce chemical dependence. Organic viticulture has emerged as one possible response to these demands. It is a production system governed by regulatory frameworks that prohibit synthetic chemical inputs, including pesticides, herbicides, and soluble mineral fertilizers, in favor of natural and biological alternatives aimed at maintaining soil health, biodiversity, and ecological balance within the vineyard^27,28^. However, it often relies on repeated applications of copper- and sulfur-based products with attendant risks of soil contamination^29^ and may face limitations under high disease pressure. In this context, the integration of disease-resistant varieties combined with decision support-based IPM strategies can provide practical pathways toward more resilient viticultural systems^9,30^.

To meet these challenges, a paradigm shift is needed, moving from reactive chemical protection to the proactive building of biological and genetic resilience. Two recent European initiatives, funded under the call “HORIZON-CL6-2023-BIODIV-01-14: Biodiversity friendly practices in agriculture - breeding for Integrated Pest Management (IPM)”: Shield4Grape (https://shield4grape.eu/) and GrapeBreed4IPM (https://grapebreed4ipm.com/) exemplify how these directions are being operationalized through collaborative research. Together, these projects advance five interconnected pillars that we argue must underpin the viticulture of the future: (1) expanding grapevine genetic diversity through traditional breeding^9^; (2) accelerating genetic improvement via new genomic techniques (NGTs)^31–34^; (3) harnessing somaclonal plasticity for adaptive responses^35^; (4) embedding these innovations within cultivar-tailored, knowledge-driven integrated pest management strategies (IPM)^36^, and (5) evaluating socioeconomic and policy dimensions of these approaches^37,38^.

Although this paper focuses primarily on wine viticulture (the production of grapes for wine), it is worth noting that table grape production faces analogous challenges in terms of disease pressure, climate change impacts, and the need to reduce pesticide use. The genetic approaches, breeding schemes, and IPM strategies discussed here are also relevant to table grapes, which stand to benefit directly from the advances made in wine grape research and innovation.

## Expanding the genetic base: diversity as the foundation of resilience

Decades of selection for specific enological traits have favored a handful of *V. vinifera* cultivars that define the global wine market, yet this uniformity leaves the crop vulnerable to emerging diseases and climatic extremes^39^. This vulnerability is becoming increasingly evident under ongoing global change, as rising temperatures, altered precipitation patterns, water scarcity, and more frequent late-spring frosts, heatwaves, hailstorms, and intense rainfall events are already affecting grapevine productivity, yield stability, and fruit composition, while also intensifying pest and disease pressure^23,40^. Across established wine regions, growing-season temperatures have risen substantially over recent decades, and harvest dates in major French regions such as Bordeaux and the Loire have advanced by approximately two weeks relative to the long-term historical record, with a phenological sensitivity of about six days earlier per °C of warming^41^. Under high-emission scenarios, continental-scale modeling for Europe projects warming of up to 4 °C across large parts of the continent by 2070, mean budburst and harvest dates advancing by more than one month (with regional advances of up to 30 days for budburst and up to 40 days for harvest in northern Iberia, southern France, Italy, and parts of eastern Europe), and yield decreases of up to 8 t ha⁻¹ in southern Iberia, parts of Italy, and the Aegean Sea by 2041–2070^42^. This thermal shift uncouples sugar accumulation from secondary-metabolite biosynthesis, raising berry sugar concentration (and therefore potential alcohol) while reducing titratable acidity, anthocyanin content, and varietal aroma compounds, with documented declines in wine balance and typicity in several traditional regions^40^. At the global scale, around 56% of current wine-growing regions could become climatically unsuitable for their incumbent cultivars at 2 °C of warming and around 85% at 4 °C; deploying a more diverse set of varieties more than halves these losses at 2 °C (from 56% to 24%) and reduces them by approximately one third at 4 °C (from 85% to 58%)^43^. Water scarcity is becoming the dominant constraint in Mediterranean and semi-arid regions: under high-warming scenarios, about 90% of traditional coastal and lowland wine regions of Spain, Italy, Greece, and southern California are projected to be at risk of disappearing by the end of the century because of excessive drought and more frequent heatwaves^40^. In Spain alone, vineyard area under irrigation has already risen to approximately 50% over the past few decades, with documented groundwater overuse and soil salinization in vulnerable basins, signaling that compensating water deficits with irrigation is approaching its sustainability limits^44^. Extreme weather events such as late-spring frosts, heatwaves, hailstorms, and intense rainfall are increasing in frequency and intensity and can cause regional yield losses in affected vintages^45,46^. Climate change is also reshaping disease epidemiology: warmer winters and altered humidity regimes are extending the active season of *Plasmopara viticola* and *Erysiphe necator*, increasing the number of infection cycles per growing season and shifting outbreaks to higher elevations and latitudes^47,48^, while emerging insect pests such as *Lobesia botrana* and *Scaphoideus titanus* are colonizing formerly unaffected areas^49^. Together, these compounding biotic and abiotic pressures translate into rising agrochemical inputs, elevated greenhouse-gas footprints per unit of wine produced, and a measurable erosion of the long-term sustainability of viticultural systems^20,21,40^.

Expanding the genetic diversity available for breeding is therefore a critical prerequisite for enhancing resilience. In this context, regions such as the Caucasus and West Asia, recognized as primary centers of domestication and diversification of the genus *Vitis*, represent key reservoirs of allelic diversity and locally adapted genotypes that remain largely underutilized in modern breeding programs^2^. Beyond cultivated diversity, wild grapevine accessions constitute an additional and largely untapped source of genetic variation^50^. Rather than serving solely as donors of major resistance loci, these populations provide access to complex and geographically structured genetic architectures shaped by long-term local adaptation. In particular, wild populations from regions such as the Balkans, the Caucasus, and Central Asia harbor unique allelic combinations that can inform the identification of resilient genetic patterns^51^. Their systematic incorporation into breeding programs could therefore enhance the development of more robust and adaptable cultivars^4^. At the same time, the strong reliance on a limited number of *V. vinifera* cultivars constrains the adaptive capacity of viticulture systems. This climatic and phytosanitary trajectory exposes a structural vulnerability of modern viticulture. The elite *V. vinifera* cultivars that dominate global plantings were selected over the past two centuries under climatic and pathogen-pressure regimes that no longer prevail^39,52^. Premium wine production has been associated with relatively narrow, cultivar-specific growing-season climatic envelopes, and warming beyond these envelopes is consistently associated with reduced berry quality, accelerated sugar accumulation, and physiological dysfunction^39,40^. Elite *V. vinifera* cultivars are also intrinsically susceptible to powdery and downy mildews and to grapevine trunk diseases, requiring repeated fungicide applications to maintain commercial yields^11,13,52^. The global rootstock pool is similarly constrained: virtually all rootstocks in commercial use derive from interspecific hybrids of three North American species (*V. riparia*, *V. rupestris*, and *V. berlandieri*), originally selected at the end of the 19th century for resistance to phylloxera and adaptation to the soils of central and southern Europe; their performance under the combinations of drought, salinity, waterlogging, and heat stress now expected under climate change is generally suboptimal, and breeding objectives for next-generation rootstocks remain only partially defined^26,53,54^. As a consequence, both the scion and the rootstock components of the global vineyard rest on a genetic base that is too narrow, too specialized, and too historically anchored to absorb the magnitude and pace of current global change without targeted innovation.

To control PM and DM with minimal use of agrochemicals, genetic improvement through the interspecific introgression of resistance genes (*R* genes) has been the most straightforward approach^55,56^. A few factors conferring protection against PM and DM have been identified in grapevine, while several others are known as quantitative trait loci (QTLs) and the underlying resistance haplotypes are introgressed as extended chromosomal segments^57,58^. Most donors of these factors are wild relative species, with a few exceptions in *V. vinifera* germplasm^59–61^. Gene pyramiding, the combination of multiple *R* genes or haplotypes, is expected to provide broader and more durable resistance to pathogens^62^. Although traditional crossbreeding is effective in creating varieties, particularly through Marker-Assisted Selection (MAS), it is time-consuming, constrained by available genetic variation, and phenotypic selection is labor-intensive for complex traits. In this context, the increasing availability of next-generation genomic tools, including whole-genome sequencing (WGS) and transcriptomics, is profoundly reshaping grapevine research and breeding. Compared with traditional marker-based approaches, which target a limited number of polymorphic loci, these high-throughput technologies enable genome-wide resolution of genetic variation and gene expression dynamics. This allows a more comprehensive dissection of complex traits, including quantitative disease resistance and stress adaptation, and supports the transition toward predictive breeding strategies^63^. Expanding genetic diversity is not only a strategy to enhance resistance to individual stresses but a prerequisite for developing cultivars capable of coping with the interacting drivers of global change^64^. These threats vary across spatial scales and demand a hierarchical innovation agenda. At the global scale, the priority is to broaden the breeding base by mobilizing heat- and drought-tolerant genetic resources from underused *V. vinifera* germplasm and from wild *Vitis* species^2,4,39,43^. At the regional scale, breeding priorities diverge sharply: Mediterranean, semi-arid, and emerging subtropical regions urgently require water- and heat-resilient cultivars and rootstocks, whereas Atlantic and continental regions benefit most from durable multi-locus disease resistance and from cultivars phenologically buffered against compressed ripening and late-spring frost^23,40,41,45^. At the local scale, terroir-specific clones, somaclones, and varieties are essential to preserve appellation identity while improving resilience^22,23,65,66^. Among the drivers of global change, water scarcity, heat stress, and the intensification of fungal disease cycles most urgently demand cultivar innovation, with extreme events acting as compounding stressors that require phenological tuning alongside canopy management^45,46,66^.

Over the past two decades, genomic selection (GS) has emerged as a powerful method to enhance breeding efficiency and reduce the time required to select for complex traits^67^. In grapevine, GS has recently been applied to several traits related to berry composition and yield components^68,69^. However, its broader implementation for predicting the overall breeding value of individual seedlings and its integration into routine breeding pipelines still require substantial research. Advancing GS methodologies for viticulture will strengthen conventional crossbreeding by facilitating the integration of multiple traits and enabling the early evaluation of a much larger number of seedlings prior to field testing, thereby accelerating genetic gain and shortening breeding cycles.

**Fig. 2:**
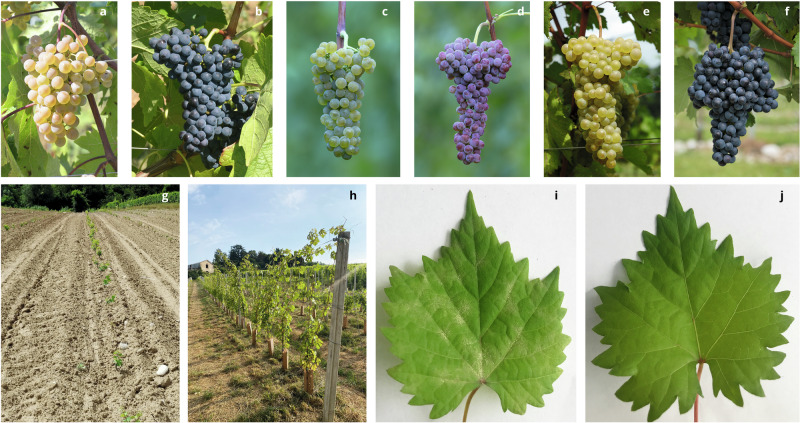
Illustration of disease-resistant varieties (DRVs), somaclones and edited plants for resistance against powdery mildew. Pictures in **a**–**f** show some of the current DRVs developed in European breeding programs with resistance against DM and PM: **a** Opalor and **b** Sirano from the INRAE-ResDur program (France); **c** Solaris and **d** Souvignier gris from the Staatliches Weinbauinstitut Freiburg breeding program (Germany); **e** Soreli and **f** Pinot Kors from Vivai Cooperativi Rauscedo (VCR, Italy). **g**, **h** show somaclones grafted onto SO4 and self-rooted somaclones respectively; **i**, **j** show leaves from a PM-infected control plant and from an edited plant targeting miRNA482 to induce resistance to the pathogen.

The resulting disease-resistant varieties (DRVs) obtained by traditional breeding (see Fig. 2 for some examples), often referred to as PIWI varieties (from the German *Pilzwiderstandsfähige Rebsorten*, “fungus-resistant grape varieties”) in several EU countries, offer remarkable reductions in fungicide use by up to 80% compared with traditional cultivars^70^. Although countries such as France, Germany, Italy, Spain, and Switzerland have established leading research and breeding programs on grapevine resilience, the absence of a coordinated European framework limits their collective impact. A concerted transnational effort is essential to scale up innovation and address continental challenges in a coherent manner^38,71^. Adoption of DRVs remains highly heterogeneous across European wine-producing regions. While countries such as Germany, Switzerland or the Czech Republic have embraced these innovations most actively^72^, others, such as Italy, show a more limited inclination toward varietal renewal^73^. Despite recent progress, DRVs currently represent less than 1% of the vineyard area within the EU. Importantly, policy evolution is beginning to align with innovation: Regulation (EU) 2021/2117 now allows hybrid grape varieties in Protected Designation of Origin (PDO) and Protected Geographical Indication (PGI) labels, signaling recognition that environmental sustainability is compatible with cultural heritage. However, DRV genotypes currently available for planting in Europe were selected in the past under scenarios of disease pressure, climate conditions and expectations for the attainable level of pesticide reduction, factors that have now significantly changed. Changing disease pressures, stricter pesticide regulations, and evolving climate conditions require a new generation of DRVs with broader and more durable resistance profiles.

## New genomic techniques

Traditional breeding, while powerful, is inherently slow. Grapevines are long-lived, highly heterozygous, and propagated vegetatively, making each breeding cycle costly in both time and resources. New genomic techniques (NGTs), notably cisgenesis and targeted genome editing, offer a route to accelerate improvement without compromising varietal integrity^74,75^. Transgenic approaches, which involve interspecies gene transfer, face strong regulatory and societal resistance in the EU. In contrast, cisgenesis and genome editing operate within the species’ natural gene pool or target endogenous sequences. Cisgenesis enables the transfer of specific genes between interfertile species while preserving the original genotype and phenotypic identity, thereby avoiding the extensive genetic reshuffling inherent to conventional crossbreeding^76,77^. Genome editing precisely modifies or silences genes associated with undesirable traits^31,78–80^. Both methods can shorten breeding cycles, and the European Food Safety Authority (EFSA) has assessed several NGT-derived changes as posing no new hazards compared with comparable products of conventional breeding. These approaches are particularly valuable in premium wine regions, where maintaining historical cultivars and wine typicity remains essential.

Despite their potential, several limitations currently constrain the effective deployment of NGTs in grapevine improvement. Transformation and regeneration efficiencies remain low and highly genotype-dependent, with strong variability among cultivars that limits the transferability of protocols across genetic backgrounds. This cultivar-specific response represents a major bottleneck for the application of genome editing in elite varieties^81^. In addition, many traits relevant to climate adaptation, such as drought tolerance, heat resilience, and yield stability, are polygenic and strongly influenced by genotype-by-environment (G × E) interactions, making them difficult to manipulate through single-gene editing strategies. Economic and infrastructural barriers further restrict the adoption of NGTs. High development costs, the need for specialized facilities, and unequal access to genomic resources and technical expertise across regions create disparities between well-funded research systems and emerging viticultural areas, potentially limiting the global scalability of these approaches^82^. At the same time, societal acceptance remains a critical factor. While cisgenesis and genome editing are often perceived as more acceptable than transgenic approaches, consumer awareness of these technologies is still limited, and skepticism toward biotechnology in agriculture persists, particularly in traditional wine-producing regions^37^.

The development of NGT-derived plants should be accompanied by comprehensive assessments, including whole-genome sequencing before and after editing, to investigate any unintended variations. Such rigorous validation provides transparent evidence of the precision and control of these techniques, thereby informing policymakers, producers, and consumers. Recent EU policy developments point toward risk-proportionate regulation of NGTs. If fully adopted, this framework would classify certain genome-edited plants as equivalent to conventionally bred varieties, provided the resulting genetic changes could also occur naturally or through conventional breeding. This evolution in regulation would enable both public and private breeders to respond more rapidly to emerging disease threats and climate challenges.

## Somaclonal plasticity: beyond the genome

Somatic embryogenesis occurs spontaneously in some plant species and can be induced in tissue cultures of other species, during which stressful culture and regeneration conditions can trigger genetic or epigenetic variation (somaclonal variation)^81,83^. Although somaclonal variation was first observed more than 40 years ago in plant cells grown in vitro^84^, few examples of practical applications in plant breeding are available to date. Currently, somaclones (grapevine somatic mutants obtained through somatic embryogenesis) are being produced by eliciting explants grown in vitro with resistance inducers^35^. This early priming enhances the activation of plant defense mechanisms, shifting physiological trade-offs toward increased tolerance to a wide range of stresses^85^. Primed grapevine somaclones with improved adaptation to biotic and abiotic stresses are currently being evaluated under greenhouse and field conditions^35^. This represents a promising route for viticulture, with potential short-term benefits for both growers and industry. Unlike NGTs, this genetic improvement strategy does not require prior knowledge of the genetic determinants of the trait of interest, making it particularly valuable for complex, polygenic, and environmentally influenced traits. These resilient genotypes, developed within the genetic background of traditional cultivars, could provide a deployable option for vineyards. In parallel, clonal diversity within existing grapevine cultivars represents a complementary and immediately deployable source of adaptive variation^86^. Grapevine is predominantly propagated vegetatively, and centuries of clonal selection have generated substantial intra-varietal diversity^87^, often associated with differences in phenology, productivity, berry composition, and responses to environmental stresses. This diversity reflects the accumulation of somatic mutations and epigenetic variation over time, resulting in distinct clones within the same cultivar^88^. Clonal selection can be directly implemented within existing regulatory frameworks and production systems, while preserving varietal identity and wine typicity^89^. The combined use of somaclonal variation and clonal selection can expand the adaptive potential of grapevine by leveraging both induced and naturally occurring variation, offering a scalable and socially accepted pathway toward more resilient viticultural systems.

Table 1 summarizes the complementarity among breeding techniques currently available in Vitis spp. Such a multi-approach strategy maximizes the potential to deliver new genotypes with enhanced tolerance to biotic and abiotic stresses across viticultural systems.

**Table 1:** Features of the different genetic improvement approaches available in grapevine

Disease resistant varieties (DRVs)	New genomic techniques (NGTs)	SOMACLONES
New varieties are obtained, with potential changes in sensory quality relative to parental *V. vinifera* lines	Traditional grape varieties are maintained, and wine identity should be preserved; field validation is needed	Traditional grape varieties are maintained, and wine identity should be preserved; field validation is needed
Long breeding cycles (12-15 years) and high costs	Reduced breeding time (5-8 years), but relatively high development and regulatory costs	Reduced breeding time (5-8 years) and moderate costs
Introgression of resistance genes against specific pathogens. Possibility to combine traits (resistance, productivity, quality)	Possibility of inducing broad-spectrum tolerance to different pathogens	Possibility of inducing broad-spectrum pathogen tolerance or multistress tolerance
Potential emergence of pathogen strains able to overcome introgressed resistance. Combining multiple resistance sources is essential.	The emergence of resistance-breaking strains may be reduced if a broad-spectrum tolerance is achieved; field validation is needed	The emergence of resistance-breaking strains may be reduced if a broad-spectrum tolerance is achieved; field validation is needed
Prior knowledge of the genetic basis of the trait of interest is needed	Prior knowledge of the genetic basis of the trait of interest and precise target gene(s) is needed	Prior knowledge of the genetic basis of the trait of interest is not required
Poor knowledge of genetic traits associated with different biotic and abiotic stress tolerance	Multi-target genome editing is under development	Useful for those traits relying on complex genetic bases and highly influenced by environmental conditions, such as those associated with abiotic stress tolerance
Subject to strict regulatory frameworks related to wine production under Protected Designation of Origin (PDO) labels	Subject to the forthcoming EU regulatory framework for NGTs, expected to apply from 2028	Generally compatible with current clonal registration pathways, subject to national variety and plant material regulations

## Cultivar-tailored integrated pest management strategies

No genetic innovation can succeed without appropriate management. Integrated pest management (IPM) remains the operational framework through which sustainability becomes tangible. In grapevine, IPM must evolve from generalized recommendations to cultivar-specific strategies that reflect each genotype’s strengths and vulnerabilities. Such precision IPM integrates multiple data sources: disease surveillance, weather and microclimate monitoring, pathogen population genomics, and decision support systems (DSS). For DRVs, the aim is to define intervention thresholds that minimize pesticide use while slowing resistance erosion. DSS models can guide growers in identifying when interventions are truly necessary, reducing prophylactic spraying and aligning treatments with pathogen life cycles. However, the effectiveness of these approaches depends on their adoption by growers, which varies widely according to farm size, technical capacity, and access to advisory services. While DSS tools have demonstrated their potential to reduce pesticide use and improve decision-making, their implementation in practice may be constrained by data availability, digital infrastructure, and the need for user-friendly interfaces and training^90^.

Long-term success depends on resistance stewardship. The deployment of DRVs across viticultural landscapes must be strategically managed to avoid uniform selection pressure that accelerates pathogen adaptation. Maximizing the durability of resistance requires continuous monitoring of pathogen virulence dynamics. A Europe-wide network of vineyard plots planted with DRVs is currently being established to monitor DM virulence, building on the proven OSCAR network initially developed in France^70^. This expansion will encompass diverse viticultural systems and pedoclimatic contexts, enabling a more comprehensive evaluation of resistance durability. Candidate resistance-breaking isolates identified through this network will be phenotyped for virulence against different resistance genes, while population-level molecular surveillance will rely on genome sequencing of pathogen samples collected from the network. Together, these approaches will characterize the performance of contrasting IPM and organic systems and identify those that best sustain resistance over time.

Combining improved grapevine genotypes with biological control agents and/or elicitors can further reduce dependence on synthetic inputs. Beneficial microbes may be applied to prime grapevine defense responses against pathogens and environmental stresses^91^. Priming is a physiological process in which defense mechanisms are pre-activated, allowing a faster and more efficient reaction to subsequent stress. Some microorganisms can prime host defenses while also serving as direct antagonists of pathogens^92^. The practical implementation of these solutions also depends on their compatibility with existing vineyard practices and the technical capacity of growers. The use of microbial inoculants and priming agents requires clear protocols regarding timing, formulation, and environmental conditions, as well as evidence of consistent field performance^92^. Adoption is more likely when these tools are integrated into existing management routines and supported by extension services, demonstration trials, and decision support systems that translate scientific knowledge into practical guidelines.

Building on a holistic view of the plant and its microbiome, the hologenome concept defines the host and its associated microorganisms as a single functional genetic unit^93^. Advances in microbial ecology and biotechnology now allow the engineering of the plant hologenome through the design of tailored synthetic microbial communities (SynComs) to enhance disease resilience^91^. Integrating these microbiome-based innovations with genetic and agronomic strategies will accelerate the transition towards viticulture systems with lower chemical inputs and enhanced ecological sustainability. Their practical impact, however, will depend on demonstrating consistent field performance across cultivars and environments, developing stable formulations and delivery protocols, and clarifying regulatory pathways for microbial consortia.

## Socioeconomic and policy dimensions: from technology to adoption

Innovation in viticulture is not solely a scientific challenge but also a social transformation. The grape and wine sector, particularly in Europe’s traditional “Old World” regions, is deeply rooted in heritage and cultural identity, often generating resistance to technological change. Analyses of innovation dynamics indicate that new products and practices are only likely to be adopted when they emerge from co-constructed solutions and are supported by strong market demand^94–96^. Currently, there is a limited pan-European understanding of how biodiversity measures, producer initiatives, and sustainability efforts influence consumer choices, particularly regarding DRVs. Wine marketing increasingly depends on innovation and multi-channel strategies, requiring producers to adapt and negotiate with retailers, whose influence is growing^97,98^. Despite increasing consumer demand for environmentally friendly wines, adoption of sustainable production systems remains constrained by profitability concerns and implementation barriers. Within this context, IPM remains a cornerstone but requires cross-border cooperation and coordination among all actors in the value chain^99–101^.

In many traditional wine regions, appellation systems such as Protected Designations of Origin (PDOs) play a central role in shaping varietal choices and production practices^102^. These regulatory frameworks, while essential for preserving typicity and cultural heritage, can also constrain the adoption of innovative plant material, including DRVs and NGTs. Recent regulatory developments at the European level have begun to open these systems to innovation, allowing, under certain conditions, the inclusion of DRVs within PDO schemes. However, implementation remains heterogeneous across regions, and uncertainties persist regarding how to balance the preservation of terroir identity with the need to reduce environmental impacts and enhance resilience to climate change. Addressing this tension requires a re-evaluation of appellation frameworks to accommodate genetic innovation while maintaining product identity and market trust. New grapevine varieties often face challenges in market acceptance due to limited communication and consumer awareness of their benefits. However, recent studies reveal strong consumer interest in DRVs, particularly for their contribution to reducing pesticide use. Several DRV wines have been rated as equal or superior in quality to traditional varieties^73,103–105^, suggesting that sensory quality concerns may be less limiting than often assumed^103^. Nonetheless, skepticism and ethical concerns regarding agricultural biotechnology persist^82^. Few studies have assessed the perception and acceptability of NGT-derived plants among farmers and consumers, highlighting low levels of awareness and understanding^37,106^.

Participatory approaches are therefore essential to bridge these gaps. Co-design frameworks that engage farmers, breeders, advisors, and policymakers from the early stages of innovation foster ownership, trust, and practical relevance. Demonstration vineyards, field days, and participatory tasting panels can effectively communicate the agronomic, environmental, and sensory benefits of new cultivars, facilitating their broader acceptance and adoption.

## Perspective: toward a resilient and regenerative viticulture

A decade from now, the contours of a transformed viticulture could be visible across Europe and beyond. Vineyards may form dynamic mosaics of resistant varieties, locally adapted, periodically renewed, and strategically combined to stay ahead of evolving pathogens. Iconic *V. vinifera* cultivars could coexist as genome-edited lines or somaclones, maintaining their sensory identity while drastically reducing pesticide dependency. Low-impact biocontrol agents and tailored SynComs might be routinely applied at planting or pruning, reinforcing plant health and sustaining soil biodiversity. DSS tools will likely integrate real-time climatic, genomic, and microbiome data to guide precise and minimal interventions. Pan-European observatories will monitor pathogen evolution, enabling adaptive management of resistance durability. Consumers, informed by transparent labeling and certification, will increasingly recognize wines not only for their origin and flavor but also for their contribution to environmental stewardship. Nevertheless, several factors may slow this transition. In established wine regions, institutional frameworks, market expectations, and strong varietal traditions can limit the uptake of novel plant material. In addition, the longevity of vineyards and the capital investment required for replanting constrain the speed at which innovations can be introduced^13^. Practical implementation may also be uneven. Access to digital tools, technical support, and specialized knowledge differs widely among producers, and the performance of new solutions remains difficult to anticipate under variable environmental conditions. Finally, regulatory pathways and consumer attitudes will influence how rapidly these innovations can be integrated into production systems. Aligning technological advances with policy frameworks and market expectations will be essential to ensure their effective deployment^38^.

This vision does not imply homogenization or technological determinism. On the contrary, it embraces diversity (genetic, ecological, and cultural) as the essence of resilience. In this framing, genomics and biotechnology become instruments to safeguard, rather than replace, the distinctiveness of terroir. By aligning scientific innovation with participatory governance and ethical responsibility, viticulture can pioneer the broader transition toward regenerative and climate-resilient agriculture.

## Concluding remarks

Viticulture exemplifies both the challenges and the opportunities of agriculture in a changing climate. Its deep cultural heritage, high economic value and disproportionate environmental footprint make it simultaneously a symbol and a testing ground for sustainable innovation. Building resilience will require an integrated strategy that unites genetic diversity, advanced breeding, NGTs, microbiome engineering, epigenetic and somaclonal plasticity, and cultivar-specific IPM. These are not isolated solutions but interconnected levers within a systems framework linking molecular biology, agronomy, policy, and market dynamics.

The transition will not be straightforward. Success depends as much on governance, communication, and education as on scientific and technological progress. Resistance genes can be pyramided, genomes edited, and SynComs assembled, but without coordinated stewardship, societal acceptance, and supportive policy, their impact will remain limited. Conversely, when these components align, the potential is transformative: reduced chemical inputs, restored biodiversity, stabilized yields, and preserved wine quality. In this integrated vision, the vineyard becomes a living laboratory, where innovation and heritage coexist in a dynamic balance. As the EU seeks to position itself as a global leader in sustainable agriculture, initiatives such as Shield4Grape and GrapeBreed4IPM mark a shift toward participatory and science-based innovation in viticulture, combining cutting-edge genetic tools with sustainable farming practices and active engagement across the entire value chain. Viticulture, at the crossroads of tradition, science, and artistry, is uniquely positioned to demonstrate that sustainability and excellence are not opposing ideals but mutually reinforcing goals. The lessons drawn from this transformation will extend well beyond the vineyards, offering a blueprint for other perennial crops and for agriculture more broadly: resilience must be designed, shared, governed, and sustained.

## Data Availability

No datasets were generated or analyzed during the current study.
